# A Comparative Study of Carotid Ultrasound Findings in Patients With and Without Familial Hypercholesterolemia

**DOI:** 10.7759/cureus.92429

**Published:** 2025-09-16

**Authors:** Rina Nakajima, Hiroki Yamazaki, Kayoko Kozuma, Takashi Suzuki, Kenji Uno, Koji Morita, Toshio Ishikawa, Satoshi Miyata, Tamio Teramoto, Kazuhisa Tsukamoto

**Affiliations:** 1 Division of Endocrinology and Metabolism, Department of Internal Medicine, Teikyo University School of Medicine, Tokyo, JPN; 2 Teikyo Academic Research Center, Teikyo University, Tokyo, JPN; 3 Public Health, Teikyo University Graduate School of Public Health, Tokyo, JPN; 4 Clinical Research Medicine Course, Teikyo University, Tokyo, JPN

**Keywords:** atherosclerosis, carotid ultrasound, familial hypercholesterolemia, intima-media thickness, plaque score

## Abstract

Background and purpose: Familial hypercholesterolemia (FH) is a genetic disorder that can lead to coronary artery disease and heart valve disease at a young age. Carotid ultrasound is a relatively simple examination for evaluating atherosclerosis. This study aimed to clarify the differences in carotid ultrasound findings between FH and non-FH patients and to identify the characteristics of atherosclerosis in FH.

Subjects and methods: Patients with FH or non-FH who underwent carotid ultrasound at a dyslipidemia clinic were enrolled. Background information and clinical laboratory values were extracted at the time of the initial ultrasound examination. Background factors were matched, and carotid ultrasound findings (plaque score (PS) and intima-media thickness (IMT)) were compared between the two groups.

Results: The FH group exhibited significantly higher levels of PS and IMT than the non-FH group. In those under 50 years of age, no significant differences in PS or IMT were observed between the FH and non-FH groups. On the other hand, in those aged 50 years or over, the FH group exhibited significantly higher levels of PS and IMT than the non-FH group. There were no significant differences in the changes and change rates in PS and IMT over time between the two groups.

Conclusion: The importance of early diagnosis and adequate treatment of FH was suggested in FH management.

## Introduction

Familial hypercholesterolemia (FH) is a genetic disorder characterized by elevated serum low-density lipoprotein cholesterol (LDL-C) levels from childhood, leading to coronary artery disease and valvular heart disease at a young age. Therefore, treatment should be initiated as early as possible. Due to the absence of symptoms, however, it often goes undetected in the early stages, and some cases remain undiagnosed until coronary artery disease develops. Additionally, there are cases where FH is not diagnosed even after the onset of atherosclerotic diseases, and active lipid-lowering therapy is not administered.

Carotid ultrasound is a relatively simple test for evaluating atherosclerosis. It evaluates intima-media thickness (IMT) and plaque score (PS), both of which are associated with an increased risk of myocardial infarction and stroke [1]. Therefore, IMT and PS are used as surrogate markers for atherosclerotic diseases. Even in studies focusing solely on FH, carotid PS has been shown to correlate with coronary PS and major cardiovascular events [2].

FH is associated with faster progression of atherosclerosis than non-FH, and differences in carotid ultrasound findings have been reported between the two groups [3]. For example, a study comparing carotid ultrasound findings in FH and non-FH patients under 30 years of age reported significantly higher PS in FH compared to non-FH [4]. Additionally, a comparative study between FH and their first-degree relatives without FH reported significantly higher maximum IMT and PS in FH [5]. However, IMT and PS are influenced by numerous factors other than the genetic background of FH, including age, sex, presence of diabetes, and presence of hypertension [6]. Therefore, to understand the characteristics of atherosclerosis in FH, research designs that account for the influence of other atherosclerosis-promoting factors are necessary. Previous studies comparing FH and non-FH patients have not always sufficiently considered this aspect, and there are few reports examining differences in carotid ultrasound findings or their longitudinal changes between FH and non-FH groups based on factors such as gender, age, body mass index (BMI), comorbidities, treatment status, or LDL-C levels. In other words, the detailed characteristics of carotid ultrasound findings in FH remain unclear, and there is a lack of basic knowledge regarding patient background factors that should be considered in the prevention and management of atherosclerosis in FH.

In this study, we collected data on FH and non-FH patients from one facility and matched their background information. We analyzed the differences and time-dependent changes in carotid ultrasound findings between FH and non-FH patients. The aim of this study was to identify the characteristics of atherosclerosis and factors associated with atherosclerosis progression in FH.

## Materials and methods

This study is a single-center retrospective observational study. Patients who visited a dyslipidemia clinic in Tokyo were enrolled, and their background information and clinical laboratory values were collected. The study was conducted with the approval of Teikyo University Medical Research Ethics Committee (approval number: 24-084).

Study population

The study included cases that met both of the following criteria: 1) patients who visited the dyslipidemia clinic and met the Japan Atherosclerosis Society diagnostic criteria for FH at the time of diagnosis and underwent carotid ultrasound examination at the clinic, or patients who did not meet the diagnostic criteria for FH but underwent carotid ultrasound examination, and 2) patients who were 18 years of age or older at the time of carotid ultrasound examination [7,8]. From the 84 FH cases and 156 non-FH cases, propensity scores for FH and non-FH were calculated using logistic regression analysis, with age, sex, and the presence or absence of diabetes, hypertension, coronary artery disease, and lipid-lowering medication at the time of carotid ultrasound as covariates. The matched case groups were then analyzed using the PSMACH procedure in SAS (SAS Institute, Cary, NC), version 9.4, with 1:1 matching, duplicate exclusion, and a caliper of 0.2. The initial ultrasound examination dates for patients before propensity score matching ranged from 20 July 2013 to 10 May 2024. For matched patients, the initial ultrasound examination date ranged from 11 October 2013 to 16 February 2024. To analyze time-dependent changes, data on the most recent ultrasound examination date were collected, yielding data up to 14 February 2025.

Data acquisition

Data obtained included: clinical laboratory values (total cholesterol, direct LDL-C or LDL-C calculated using the Friedewald formula, high-density lipoprotein cholesterol (HDL-C), and triglycerides) at the time of the initial carotid ultrasound examination performed at the clinic; patient background information (sex, age, and BMI); presence or absence of comorbidities (coronary artery disease, diabetes, and hypertension); presence or absence of statin use and other lipid-lowering medication for dyslipidemia.

Comorbidities were extracted from medical records or medication history. Carotid ultrasound examination data included IMT and PS. IMT was defined as the maximum IMT value in the common carotid artery. PS was calculated as the sum of the left and right IMT thickening values of 1.1 mm or more, measured 1.5 cm distally and 1.5 cm centrally from the bifurcation of the internal and external carotid arteries. In cases where carotid ultrasound examinations had been performed two or more times, IMT and PS in the most recent carotid ultrasound examination were also obtained to examine the time-dependent change and rate of change. Achilles tendon thickness data from soft X-ray examinations were also obtained if they were available.

Analysis methods

Continuous variables were presented as means ± standard deviations (SD) or medians with minimum, 25th, and 75th percentiles (Q1-Q3) and maximum. Categorical variables were presented as counts (percentages). The normality of continuous variables (PS and IMT) was assessed using the Shapiro-Wilk normality test. For comparisons between the FH and non-FH groups, continuous variables were analyzed using the Wilcoxon rank-sum test or Welch's t-test, and categorical variables were analyzed using Fisher's exact test. Subgroup analyses were performed for PS and IMT, as well as for their changes and change rates. Subgroup variables included sex, age, history of coronary artery disease, diabetes, hypertension, LDL-C levels, and HDL-C levels. Furthermore, as an exploratory evaluation, scatter plots were created for age and PS, and linear regression analyses were performed. The significance level was set at less than 5%. All analyses were performed using SAS Release 9.4.

## Results

Patient background

**Figure 1:**
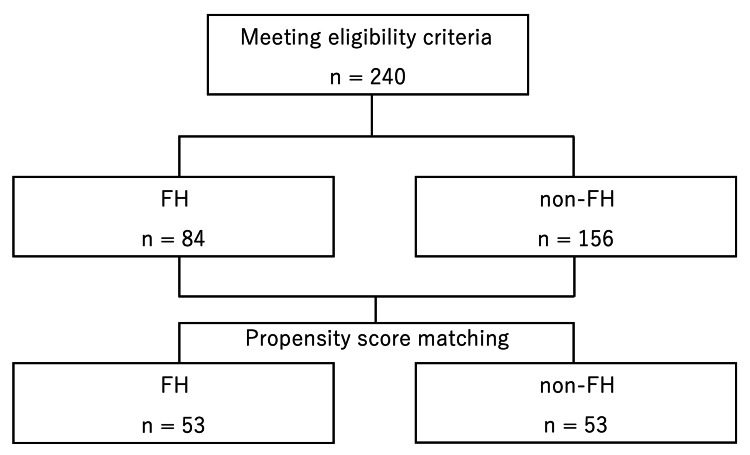
Study flow diagram FH: familial hypercholesterolemia

Eighty-four FH cases and 156 non-FH cases that met the eligibility criteria were matched using propensity score matching, and 53 matched cases from each group were analyzed (Figure 1). The patient background of all 240 cases is presented in Table 1, and comparisons of patient background before and after matching are shown in Table 2. The patient characteristics of the FH and non-FH groups after matching are shown in Tables 3-4. There were no significant differences between the FH and non-FH groups in BMI, LDL-C, HDL-C, or triglyceride levels (Table 3). Lipid-lowering medications were prescribed to 44 cases (83.0%) of the FH group and 42 cases (79.2%) of the non-FH group, and statins were administered to 43 cases (81.1%) of the FH group and 40 cases (75.5%) of the non-FH group, with no significant difference between the two groups (Table 4). The percentage of taking lipid-lowering medications other than statins, however, was significantly higher in the FH group (27 cases, 50.9%) than in the non-FH group (7 cases, 13.2%) (p < 0.001) (Table 4).

**Table 1:** Patient background at the initial carotid ultrasound, before the propensity score matching For the categorical variables, the data are represented as N (%), and the p-values were calculated by Fisher’s exact tests. For the continuous variables (age and BMI), the number of data points is presented in the upper row, and the mean ± standard deviation is presented in the lower row; p-values calculated by Welch's t-tests are shown with t-values. p < 0.05 was considered to be statistically significant. FH: familial hypercholesterolemia

Characteristic		FH (n = 84)	non-FH (n = 156)	t-value	p-value
Sex	Male	22 (26.2%)	68 (43.6%)		
	Female	62 (73.8%)	88 (56.4%)		0.008
Coronary artery disease	+	6 (7.1%)	6 (3.8%)		
	−	78 (92.9%)	150 (96.2%)		0.352
Diabetes	+	1 (1.2%)	36 (23.1%)		
	−	83 (98.8%)	120 (76.9%)		<0.001
Hypertension	+	6 (7.1%)	60 (38.5%)		
	−	78 (92.9%)	96 (61.5%)		<0.001
Smoking	+	1 (1.2%)	10 (6.4%)		
	−	83 (98.8%)	146 (93.6%)		0.103
Cholesterol-lowering medication	+	75 (89.3%)	110 (70.5%)		
	−	9 (10.7%)	46 (29.5%)		0.001
Use of statins	+	73 (86.9%)	104 (66.7%)		
	−	11 (13.1%)	52 (33.3%)		<0.001
Use of cholesterol-lowering medication other than statins	+	47 (56.0%)	28 (17.9%)		
	−	37 (44.0%)	128 (82.1%)		<0.001
Age		84	156		
— year		46.1 ± 15.4	58.9 ± 10.6	6.82	<0.001
BMI		79	154		
— kg/m^2^		22.0 ± 3.8	23.5 ± 3.4	2.87	0.005

**Table 2:** Patient background at the initial carotid ultrasound, before and after the propensity score matching For the continuous variable (age), the data are represented as mean ± standard deviation. For the categorical variables, the data are represented as N (%). FH: familial hypercholesterolemia; ASD: absolute standardized difference

Characteristic		Before Matching			After Matching		
		FH (n = 84)	non-FH (n = 156)	ASD	FH (n = 53)	non-FH (n = 53)	ASD
Age — year		46.1 ± 15.4	58.9 ± 10.6	-0.971	53.4 ± 12.0	53.4 ± 9.3	0.000
Sex	Male	22 (26.2%)	68 (43.6%)	0.371	17 (32.1%)	12 (22.6%)	-0.201
Diabetes	+	1 (1.2%)	36 (23.1%)	0.711	1 (1.9%)	1 (1.9%)	0.000
Hypertension	+	6 (7.1%)	60 (38.5%)	0.805	5 (9.4%)	6 (11.3%)	0.048
Coronary artery disease	+	6 (7.1%)	6 (3.8%)	-0.145	2 (3.8%)	1 (1.9%)	-0.083
Cholesterol-lowering medication	+	75 (89.3%)	110 (70.5%)	-0.482	44 (83.0%)	42 (79.2%)	-0.097

**Table 3:** Patient background at the initial carotid ultrasound (continuous variables), after the propensity score matching For age, BMI, LDL-C, and HDL-C, the N of data is presented in the upper row, and the mean ± standard deviation is presented in the lower row. Welch’s t-tests were performed, and p-values are shown with t-values. For TG, the N of data is presented in the upper row, and the median (minimum, the first quartile, the third quartile, maximum) is presented in the lower row. The Wilcoxon rank-sum test was performed, and the p-value is shown with the z-value. p < 0.05 was considered to be statistically significant. FH: familial hypercholesterolemia; TG: triglycerides

Characteristic	FH (n = 53)	non-FH (n = 53)	t- or z-value	p-value
Age	53	53		
— year	53.4 ± 12.0	53.4 ± 9.3	0.00	1.000
BMI	51	53		
— kg/m^2^	22.6 ± 4.2	22.5 ± 3.9	0.14	0.889
LDL-C	53	53		
— mg/dL	150.8 ± 64.2	138.2 ± 32.0	1.28	0.203
HDL-C	53	53		
— mg/dL	73.2 ± 25.0	74.5 ± 16.9	0.31	0.754
TG	53	53		
— mg/dL	102 (39, 77, 172, 3159)	106 (34, 81, 139, 647)	0.16	0.869

**Table 4:** Patient background at the initial carotid ultrasound (categorical variables), after the propensity score matching The data are represented as N (%). p-values were calculated by Fisher’s exact tests. p < 0.05 was considered to be statistically significant. FH: familial hypercholesterolemia

Characteristic		FH (n = 53)	non-FH (n = 53)	p-value
Sex	Male	17 (32.1%)	12 (22.6%)	
	Female	36 (67.9%)	41 (77.4%)	0.384
Coronary artery disease	+	2 (3.8%)	1 (1.9%)	
	−	51 (96.2%)	52 (98.1%)	1.000
Diabetes	+	1 (1.9%)	1 (1.9%)	
	−	52 (98.1%)	52 (98.1%)	1.000
Hypertension	+	5 (9.4%)	6 (11.3%)	
	−	48 (90.6%)	47 (88.7%)	1.000
Smoking	+	0 (0.0%)	1 (1.9%)	
	−	53 (100.0%)	52 (98.1%)	1.000
Cholesterol-lowering medication	+	44 (83.0%)	42 (79.2%)	
	−	9 (17.0%)	11 (20.8%)	0.804
Use of statins	+	43 (81.1%)	40 (75.5%)	
	−	10 (18.9%)	13 (24.5%)	0.638
Use of cholesterol-lowering medication other than statins	+	27 (50.9%)	7 (13.2%)	
	−	26 (49.1%)	46 (86.8%)	<0.001

Comparison of plaque score (PS)

The comparison of PS between the FH and the non-FH group is shown in Table 5. As the Shapiro-Wilk test revealed that neither group was normally distributed (p < 0.001), nonparametric tests (Wilcoxon rank-sum tests) were used to compare the groups. The FH group had significantly higher PS than the non-FH group (FH group: 2.5 (1.1-5.0); non-FH group: 1.3 (0-2.2); p = 0.003).

**Table 5:** Comparison of PS between FH and non-FH Only cases after the propensity score matching were used. p-value calculated by the Wilcoxon rank-sum test is shown with the z-value. p < 0.05 was considered to be statistically significant. PS: plaque score; FH: familial hypercholesterolemia; Q1: the first quartile; Q3: the third quartile

	FH	non-FH	z-value	p-value
Count	53	53		
Median (Minimum, Q1, Q3, Maximum)	2.5 (0, 1.1, 5, 13.4)	1.3 (0, 0, 2.2, 15.4)	2.97	0.003

The results of the subgroup analysis according to background are shown in Table 6. In women, PS was significantly higher in the FH group (FH group: 2.75 (1.1-4.85); non-FH group: 1.3 (0-2.3); p = 0.023), and a similar trend was observed in men (FH group: 2.3 (1.2-5.2); non-FH group: 1.25 (0-1.7); p = 0.055). There was no difference in PS between the FH and non-FH groups in those under 50 years of age, but the FH group had significantly higher PS than the non-FH group in those aged 50 years or older (FH group: 4.1 (1.6-6.1); non-FH group: 1.3 (0-2.3); p < 0.001). Regarding the presence or absence of coronary artery disease, the FH group had significantly higher PS than the non-FH group in those without coronary artery disease. Similarly, the FH group had significantly higher PS than the non-FH group in those without diabetes. In the group with LDL-C <140 mg/dL, the FH group had significantly higher PS than the non-FH group (FH group: 2.7 (1.2-7.3); non-FH group: 1.3 (0-2.3); p = 0.030), but no significant difference was observed in the group with LDL-C ≥140 mg/dL. No significant difference was observed in the group with HDL-C <50 mg/dL, but the FH group had significantly higher PS in the group with HDL-C ≥50 mg/dL (FH group: 2.55 (1.1-5.0); non-FH group: 1.3 (0-2.3); p = 0.009).

**Table 6:** Comparison of PS between FH and non-FH, subgroup analysis Only cases after the propensity score matching were used. In each characteristic, the N of data is presented in the upper row, and the median (minimum, the first quartile, the third quartile, maximum) is presented in the lower row. For N=1 data, only the median is shown. For N=2 to 4 data, the median (minimum, maximum) is shown. Wilcoxon rank-sum tests were performed, and p-values are shown with z-values. p < 0.05 was considered to be statistically significant. PS: plaque score; FH: familial hypercholesterolemia

Characteristic		FH (n = 53)	non-FH (n = 53)	z-value	p-value
Sex	Male	17	12		
		2.3 (0, 1.2, 5.2, 11.2)	1.25 (0, 0, 1.7, 2.5)	1.92	0.055
	Female	36	41		
		2.75 (0, 1.1, 4.85, 13.4)	1.3 (0, 0, 2.3, 15.4)	2.27	0.023
Age	<50	22	16		
		1.25 (0, 0, 2.5, 8.6)	1.25 (0, 0, 2, 4.9)	0.47	0.638
	≥50	31	37		
		4.1 (0, 1.6, 6.1, 13.4)	1.3 (0, 0, 2.3, 15.4)	3.71	<0.001
Coronary artery disease	+	2	1		
		3.65 (1.2, 6.1)	0	0.61	0.540
	−	51	52		
		2.5 (0, 1.1, 5, 13.4)	1.3 (0, 0, 2.25, 15.4)	2.82	0.005
Diabetes	+	1	1		
		11.4	15.4		
	−	52	52		
		2.45 (0, 1.1, 4.85, 13.4)	1.25 (0, 0, 2.05, 8.4)	3.06	0.002
Hypertension	+	5	6		
		7.7 (1.1, 6.1, 11.2, 11.4)	0.55 (0, 0, 1.2, 15.4)	1.48	0.140
	−	48	47		
		2.4 (0, 1.1, 4.45, 13.4)	1.3 (0, 0, 2.3, 8.4)	2.41	0.016
LDL-C	<140	29	27		
		2.7 (0, 1.2, 7.3, 13.4)	1.3 (0, 0, 2.3, 15.4)	2.17	0.030
	≥140	24	26		
		2.4 (0, 0.55, 3.9, 8.4)	1.2 (0, 0, 1.6, 4.9)	1.83	0.067
HDL-C	<50	7	3		
		2.4 (0, 0, 6.1, 8.4)	0 (0, 1.2)	1.41	0.158
	≥50	46	50		
		2.55 (0, 1.1, 5, 13.4)	1.3 (0, 0, 2.3, 15.4)	2.61	0.009

Comparison of intima-media thickness (IMT)

The comparison of IMT between FH and non-FH is shown in Table 7. As the Shapiro-Wilk test revealed that neither group was normally distributed (p < 0.001), the Wilcoxon rank-sum tests were used for intergroup comparisons. The FH group had significantly greater IMT than the non-FH group (FH group: 0.7 (0.6-0.9); non-FH group: 0.7 (0.6-0.8); p = 0.043).

**Table 7:** Comparison of IMT between FH and non-FH Only cases after the propensity score matching were used. p-value calculated by the Wilcoxon rank-sum test is shown with the z-value.  p < 0.05 was considered to be statistically significant. IMT: intima-media thickness; FH: familial hypercholesterolemia; Q1: the first quartile; Q3: the third quartile

	FH	non-FH	z-value	p-value
Count	53	53		
Median (Minimum, Q1, Q3, Maximum)	0.7 (0.5, 0.6, 0.9, 9.4)	0.7 (0.5, 0.6, 0.8, 2.1)	2.03	0.043

The results of the subgroup analysis by background factors are shown in Table 8. The FH group had significantly greater IMT than the non-FH group in patients aged 50 years or older (p = 0.004), patients without diabetes (p = 0.034), and patients with LDL-C < 140 (p = 0.030).

**Table 8:** Comparison of IMT between FH and non-FH, subgroup analysis Only cases after the propensity score matching were used. In each characteristic, the N of data is presented in the upper row, and the median (minimum, the first quartile, the third quartile, maximum) is presented in the lower row. For N=1 data, only the median is shown. For N=2 to 4 data, the median (minimum, maximum) is shown. Wilcoxon rank-sum tests were performed, and p-values are shown with z-values. p < 0.05 was considered to be statistically significant. IMT: intima-media thickness; FH: familial hypercholesterolemia

Characteristic		FH (n = 53)	non-FH (n = 53)	z-value	p-value
Sex	Male	17	12		
		0.7 (0.5, 0.6, 1, 9.4)	0.6 (0.5, 0.55, 0.9, 1.2)	1.14	0.253
	Female	36	41		
		0.75 (0.5, 0.6, 0.9, 6.7)	0.7 (0.5, 0.6, 0.8, 2.1)	1.72	0.085
Age	<50	22	16		
		0.7 (0.5, 0.6, 0.7, 9.4)	0.6 (0.5, 0.55, 0.75, 1.2)	0.38	0.704
	≥50	31	37		
		0.9 (0.5, 0.7, 1.1, 6.7)	0.7 (0.5, 0.6, 0.8, 2.1)	2.85	0.004
Coronary artery disease	+	2	1		
		0.95 (0.9, 1)	0.5	0.61	0.540
	−	51	52		
		0.7 (0.5, 0.6, 0.9, 9.4)	0.7 (0.5, 0.6, 0.8, 2.1)	1.70	0.089
Diabetes	+	1	1		
		0.9	2.1		
	−	52	52		
		0.7 (0.5, 0.6, 0.95, 9.4)	0.7 (0.5, 0.6, 0.8, 1.7)	2.12	0.034
Hypertension	+	5	6		
		0.9 (0.8, 0.9, 1, 1.4)	0.75 (0.5, 0.5, 1.1, 2.1)	0.65	0.518
	−	48	47		
		0.7 (0.5, 0.6, 0.9, 9.4)	0.7 (0.5, 0.6, 0.8, 1.7)	1.63	0.102
LDL-C	<140	29	27		
		0.8 (0.5, 0.7, 1, 1.4)	0.7 (0.5, 0.6, 0.8, 2.1)	2.17	0.030
	≥140	24	26		
		0.7 (0.5, 0.6, 0.85, 9.4)	0.7 (0.5, 0.6, 0.8, 1.2)	0.35	0.729
HDL-C	<50	7	3		
		0.7 (0.5, 0.6, 1, 9.4)	0.6 (0.5, 0.7)	1.16	0.246
	≥50	46	50		
		0.75 (0.5, 0.6, 0.9, 6.7)	0.7 (0.5, 0.6, 0.8, 2.1)	1.80	0.072

Comparison of the time-dependent change in PS

Table 9 shows a comparison of the time-dependent changes in PS between the FH and non-FH groups, based on their first and most recent carotid ultrasound examinations performed at the clinic. As cases with only one ultrasound examination were excluded, the analysis included 44 cases in the FH group and 45 cases in the non-FH group. No significant difference was observed in the change in PS between the two groups.

**Table 9:** Comparison of the time-dependent change in PS between FH and non-FH Only cases after the propensity score matching were used. For each case, the annual change in PS was calculated by subtracting the PS value at the first ultrasound examination from the PS value at the most recent ultrasound examination and then dividing by the interval between the two ultrasound examinations. p-value calculated by the Wilcoxon rank-sum test is shown with the z-value. p < 0.05 was considered to be statistically significant. PS: plaque score; FH: familial hypercholesterolemia; Q1: the first quartile; Q3: the third quartile

	FH	non-FH	z-value	p-value
Count	44	45		
Median (Minimum, Q1, Q3, Maximum)	0.18 (-3.26, 0, 0.385, 1.47)	0.06 (-0.47, 0, 0.42, 1.01)	0.38	0.707

The results of the subgroup analysis by background factors are shown in Table 10. No significant differences were observed between the two groups for most background factors; however, in the group aged under 50 years, the change in PS was significantly greater in the FH group than in the non-FH group (FH group: 0.18 (0.04-0.43); non-FH group: 0 (0-0.34), p = 0.048).

**Table 10:** The time-dependent change in PS, subgroup analysis Only cases after the propensity score matching were used. In each characteristic, the N of data is presented in the upper row, and the median (minimum, the first quartile, the third quartile, maximum) is presented in the lower row. For N=1 data, only the median is shown. For N=2 to 4 data, the median (minimum, maximum) is shown. Wilcoxon rank-sum tests were performed, and p-values are shown with z-values. p < 0.05 was considered to be statistically significant. PS: plaque score; FH: familial hypercholesterolemia

Characteristic		FH (n = 44)	non-FH (n = 45)	z-value	p-value
Sex	Male	15	11		
		0.13 (-0.23, 0, 0.33, 0.61)	0 (-0.08, 0, 0.06, 0.71)	1.80	0.072
	Female	29	34		
		0.24 (-3.26, 0, 0.39, 1.47)	0.16 (-0.47, 0, 0.47, 1.01)	0.28	0.782
Age	<50	17	11		
		0.18 (0, 0.04, 0.43, 1.11)	0 (-0.08, 0, 0.34, 0.71)	1.98	0.048
	≥50	27	34		
		0.18 (-3.26, 0, 0.31, 1.47)	0.13 (-0.47, 0, 0.47, 1.01)	0.67	0.502
Coronary artery disease	+	2	1		
		0.255 (0, 0.51)	0	0.00	1.000
	−	42	44		
		0.18 (-3.26, 0, 0.38, 1.47)	0.08 (-0.47, 0, 0.435, 1.01)	0.27	0.784
Diabetes	+	1	1		
		-2.37	1.01		
	−	43	44		
		0.18 (-3.26, 0, 0.39, 1.47)	0.055 (-0.47, 0, 0.4, 0.97)	0.75	0.454
Hypertension	+	3	6		
		0 (-2.37, 0.81)	0.07 (0, 0, 0.34, 1.01)	0.54	0.590
	−	41	39		
		0.18 (-3.26, 0, 0.38, 1.47)	0.06 (-0.47, 0, 0.45, 0.97)	0.44	0.663
LDL-C	<140	23	23		
		0.18 (-3.26, 0, 0.46, 1.47)	0.01 (-0.15, 0, 0.45, 1.01)	0.55	0.581
	≥140	21	22		
		0.18 (-0.71, 0, 0.27, 1.11)	0.12 (-0.47, 0, 0.42, 0.97)	0.12	0.902
HDL-C	<50	6	2		
		0.04 (0, 0, 0.43, 0.46)	0.17 (0, 0.34)	0.00	1.000
	≥50	38	43		
		0.2 (-3.26, 0, 0.38, 1.47)	0.06 (-0.47, 0, 0.45, 1.01)	0.52	0.604

Comparison of the change rate in PS

Table 11 shows a comparison of the change rates in PS between the FH and non-FH groups. Cases with only one ultrasound examination and cases with an initial PS of 0 were excluded, resulting in 35 cases in the FH group and 34 cases in the non-FH group. No significant difference in the change rate in PS was observed between the two groups.

**Table 11:** Comparison of the change rate in PS between FH and non-FH Only cases after the propensity score matching were used. The change rate in PS per year in each case was calculated by dividing the annual change in PS by the PS in the initial ultrasound. The Wilcoxon rank-sum test was performed, and the p-value is shown with the z-value. p < 0.05 was considered to be statistically significant. PS: plaque score; FH: familial hypercholesterolemia; Q1: the first quartile; Q3: the third quartile

	FH	non-FH	z-value	p-value
Count	35	34		
Median (Minimum, Q1, Q3, Maximum)	0.06 (-0.24, 0, 0.15, 0.77)	0.065 (-0.21, 0, 0.24, 0.57)	0.51	0.609

The results of the subgroup analysis according to background factors are presented in Table 12. No background factors were found to significantly affect the change rate.

**Table 12:** The change rate in PS, subgroup analysis Only cases after the propensity score matching were used. In each characteristic, the N of data is presented in the upper row, and the median (minimum, the first quartile, the third quartile, maximum) is presented in the lower row. For N=1 data, only the median is shown. For N=2 to 4 data, the median (minimum, maximum) is shown. Wilcoxon rank-sum tests were performed, and p-values are shown with z-values. p < 0.05 was considered to be statistically significant. PS: plaque score; FH: familial hypercholesterolemia

Characteristic		FH (n = 35)	non-FH (n = 34)	z-value	p-value
Sex	Male	12	7		
		0.06 (-0.19, 0.015, 0.11, 0.43)	0 (-0.05, 0, 0.04, 0.44)	1.49	0.135
	Female	23	27		
		0.07 (-0.24, 0, 0.15, 0.77)	0.09 (-0.21, 0, 0.26, 0.57)	1.14	0.254
Age	<50	11	9		
		0.07 (0.01, 0.03, 0.17, 0.43)	0 (-0.05, 0, 0.09, 0.44)	1.37	0.169
	≥50	24	25		
		0.04 (-0.24, 0, 0.115, 0.77)	0.08 (-0.21, 0, 0.24, 0.57)	1.40	0.160
Coronary artery disease	+	2	0		
		0.215 (0, 0.43)			
	−	33	34		
		0.06 (-0.24, 0.01, 0.12, 0.77)	0.065 (-0.21, 0, 0.24, 0.57)	0.60	0.550
Diabetes	+	1	1		
		-0.21	0.07		
	−	34	33		
		0.065 (-0.24, 0.01, 0.15, 0.77)	0.06 (-0.21, 0, 0.24, 0.57)	0.30	0.763
Hypertension	+	3	3		
		0 (-0.21, 0.1)	0.07 (0, 0.29)	0.66	0.507
	−	32	31		
		0.065 (-0.24, 0.01, 0.15, 0.77)	0.06 (-0.21, 0, 0.24, 0.57)	0.20	0.842
LDL-C	<140	20	16		
		0.065 (-0.24, 0.005, 0.15, 0.43)	0.015 (-0.05, 0, 0.21, 0.44)	0.08	0.936
	≥140	15	18		
		0.04 (-0.13, 0, 0.12, 0.77)	0.105 (-0.21, 0, 0.26, 0.57)	0.76	0.445
HDL-C	<50	4	1		
		0.06 (0, 0.19)	0.29	1.06	0.289
	≥50	31	33		
		0.06 (-0.24, 0, 0.15, 0.77)	0.06 (-0.21, 0, 0.23, 0.57)	0.34	0.731

Comparison of the time-dependent change in IMT

Table 13 shows a comparison of the time-dependent changes in IMT between the FH and non-FH groups, based on their first and most recent carotid ultrasound examinations performed at the clinic. Cases with only one ultrasound examination were excluded, resulting in 44 cases in the FH group and 45 cases in the non-FH group. No significant difference in IMT change was observed between the FH and non-FH groups.

**Table 13:** Comparison of the time-dependent change in IMT between FH and non-FH Only cases after the propensity score matching were used. For each case, the annual change in IMT was calculated by subtracting the IMT value at the first ultrasound examination from the IMT value at the most recent ultrasound examination and then dividing by the interval between the two ultrasound examinations. The Wilcoxon rank-sum test was performed, and the p-value is shown with the z-value. p < 0.05 was considered to be statistically significant. IMT: intima-media thickness; FH: familial hypercholesterolemia; Q1: the first quartile; Q3: the third quartile

	FH	non-FH	z-value	p-value
Count	44	45		
Median (Minimum, Q1, Q3, Maximum)	0 (-3.41, 0, 0.025, 0.23)	0.01 (-0.15, 0, 0.02, 0.27)	0.68	0.495

The results of the subgroup analysis by background factors are shown in Table 14. No background factors were found to significantly affect IMT change.

**Table 14:** The time-dependent change in IMT, subgroup analysis Only cases after the propensity score matching were used. In each characteristic, the N of data is presented in the upper row, and the median (minimum, the first quartile, the third quartile, maximum) is presented in the lower row. For N=1 data, only the median is shown. For N=2 to 4 data, the median (minimum, maximum) is shown. Wilcoxon rank-sum tests were performed, and p-values are shown with z-values. p < 0.05 was considered to be statistically significant. IMT: intima-media thickness; FH: familial hypercholesterolemia

Characteristic		FH (n = 44)	non-FH (n = 45)	z-value	p-value
Sex	Male	15	11		
		0 (-3.41, 0, 0.02, 0.08)	0.01 (-0.06, 0, 0.02, 0.02)	0.46	0.648
	Female	29	34		
		0 (-1.44, 0, 0.03, 0.23)	0.01 (-0.15, 0, 0.02, 0.27)	0.54	0.592
Age	<50	17	11		
		0 (-3.41, 0, 0.02, 0.08)	0 (-0.06, -0.01, 0.02, 0.05)	0.27	0.787
	≥50	27	34		
		0 (-1.44, -0.03, 0.03, 0.23)	0.01 (-0.15, 0, 0.02, 0.27)	0.75	0.450
Coronary artery disease	+	2	1		
		0 (0, 0)	0		1.000
	−	42	44		
		0 (-3.41, 0, 0.03, 0.23)	0.01 (-0.15, 0, 0.02, 0.27)	0.60	0.549
Diabetes	+	1	1		
		0.06	-0.08		
	−	43	44		
		0 (-3.41, 0, 0.02, 0.23)	0.01 (-0.15, 0, 0.02, 0.27)	1.03	0.305
Hypertension	+	3	6		
		0 (-0.16, 0.06)	0 (-0.08, -0.03, 0.01, 0.01)	0.00	1.000
	−	41	39		
		0 (-3.41, 0, 0.02, 0.23)	0.01 (-0.15, 0, 0.02, 0.27)	0.97	0.333
LDL-C	<140	23	23		
		0 (-0.29, 0, 0.05, 0.23)	0.01 (-0.15, 0, 0.02, 0.27)	0.30	0.763
	≥140	21	22		
		0 (-3.41, 0, 0.02, 0.15)	0.01 (-0.06, 0, 0.02, 0.07)	0.89	0.375
HDL-C	<50	6	2		
		0 (-3.41, 0, 0.04, 0.08)	-0.015 (-0.03, 0)	0.71	0.478
	≥50	38	43		
		0 (-1.44, 0, 0.02, 0.23)	0.01 (-0.15, 0, 0.02, 0.27)	0.82	0.409

Comparison of the change rate in IMT

A comparison of the change rates in IMT between the FH and non-FH groups is presented in Table 15. No significant difference in the change rates in IMT was observed between the two groups.

**Table 15:** Comparison of the change rate in IMT between FH and non-FH Only cases after the propensity score matching were used. The change rate in IMT per year in each case was calculated by dividing the annual change in IMT by the IMT in the initial ultrasound. The Wilcoxon rank-sum test was performed, and the p-value is shown with the z-value. p < 0.05 was considered to be statistically significant. IMT: intima-media thickness; FH: familial hypercholesterolemia; Q1: the first quartile; Q3: the third quartile

	FH	non-FH	z-value	p-value
Count	44	45		
Median (Minimum, Q1, Q3, Maximum)	0 (-0.36, 0, 0.04, 0.23)	0.02 (-0.1, 0, 0.04, 0.33)	0.79	0.428

The results of the subgroup analysis by background factors are shown in Table 16. No background factors were found to significantly affect the change rates.

**Table 16:** The change rate in IMT, subgroup analysis Only cases after the propensity score matching were used. In each characteristic, the N of data is presented in the upper row, and the median (minimum, the first quartile, the third quartile, maximum) is presented in the lower row. For N=1 data, only the median is shown. For N=2 to 4 data, the median (minimum, maximum) is shown. Wilcoxon rank-sum tests were performed, and p-values are shown with z-values. p < 0.05 was considered to be statistically significant. IMT: intima-media thickness; FH: familial hypercholesterolemia

Characteristic		FH (n = 44)	non-FH (n = 45)	z-value	p-value
Sex	Male	15	11		
		0 (-0.36, 0, 0.02, 0.17)	0.01 (-0.06, 0, 0.03, 0.04)	0.53	0.593
	Female	29	34		
		0 (-0.26, 0, 0.04, 0.23)	0.02 (-0.1, 0, 0.04, 0.33)	0.61	0.541
Age	<50	17	11		
		0 (-0.36, 0, 0.05, 0.17)	0 (-0.06, -0.02, 0.03, 0.1)	0.34	0.731
	≥50	27	34		
		0 (-0.26, -0.03, 0.04, 0.23)	0.02 (-0.1, 0, 0.04, 0.33)	1.02	0.306
Coronary artery disease	+	2	1		
		0 (0, 0)	0	0.00	1.000
	−	42	44		
		0 (-0.36, 0, 0.04, 0.23)	0.02 (-0.1, 0, 0.04, 0.33)	0.72	0.474
Diabetes	+	1	1		
		0.07	-0.04		
	−	43	44		
		0 (-0.36, 0, 0.04, 0.23)	0.02 (-0.1, 0, 0.04, 0.33)	1.09	0.275
Hypertension	+	3	6		
		0 (-0.18, 0.07)	0 (-0.06, -0.04, 0.01, 0.02)	0.00	1.000
	−	41	39		
		0 (-0.36, 0, 0.04, 0.23)	0.02 (-0.1, 0, 0.04, 0.33)	1.06	0.288
LDL-C	<140	23	23		
		0 (-0.26, 0, 0.07, 0.23)	0.02 (-0.1, 0, 0.04, 0.33)	0.42	0.673
	≥140	21	22		
		0 (-0.36, 0, 0.04, 0.19)	0.015 (-0.06, 0, 0.04, 0.12)	0.77	0.438
HDL-C	<50	6	2		
		0 (-0.36, 0, 0.05, 0.1)	-0.03 (-0.06, 0)	0.71	0.478
	≥50	38	43		
		0 (-0.26, 0, 0.04, 0.23)	0.02 (-0.1, 0, 0.04, 0.33)	0.95	0.344

Association between Achilles tendon thickness and PS or IMT

**Figure 2:**
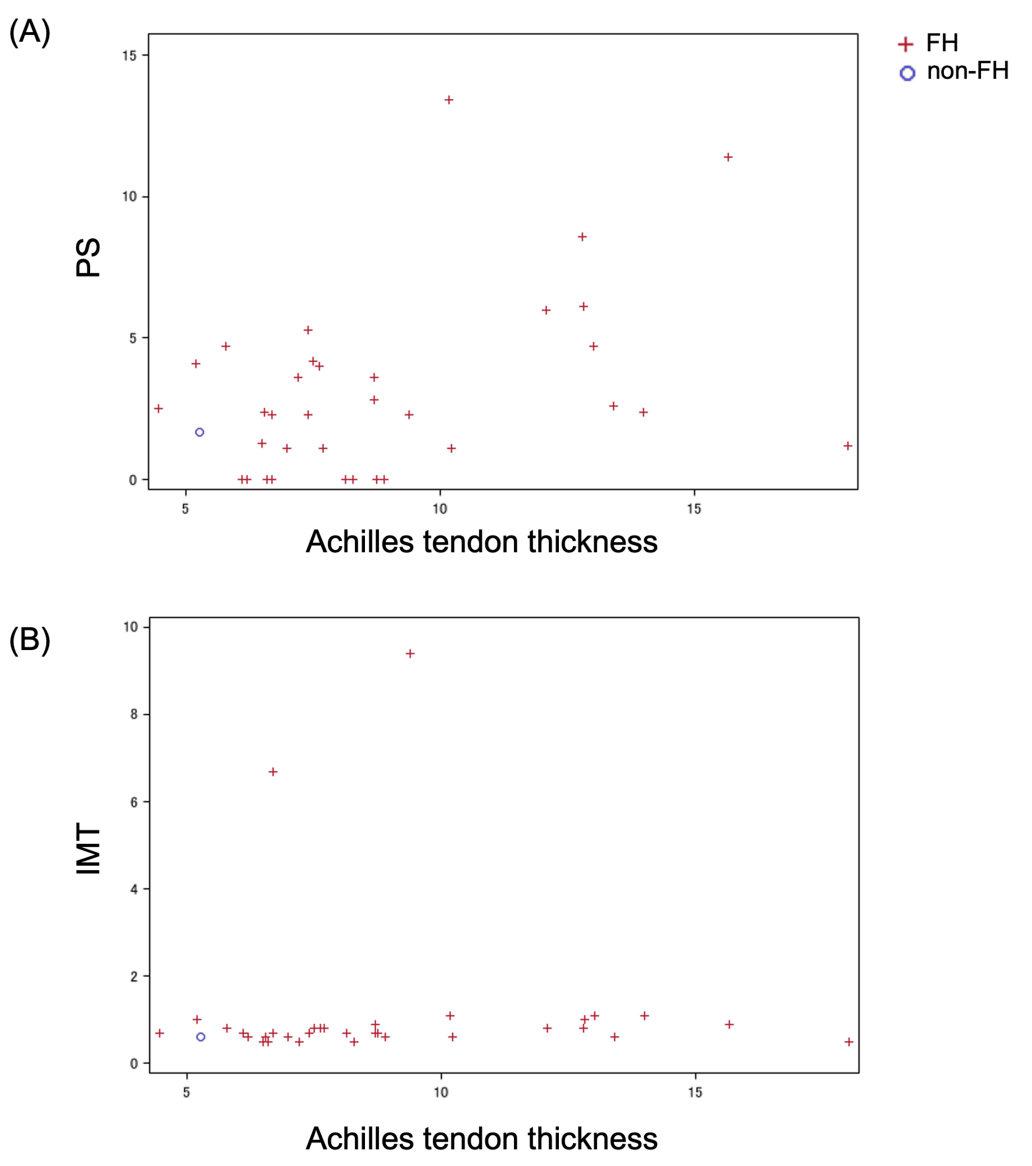
Correlation between PS or IMT and Achilles tendon thickness (A) Scatter plot of PS vs. Achilles tendon thickness. (B) Scatter plot of IMT vs. Achilles tendon thickness. FH, n = 34; non-FH, n = 1. PS: plaque score; IMT: intima-media thickness; FH: familial hypercholesterolemia

Of the cases included in this study, Achilles tendon thickness was measured in 34 cases in the FH group and one case in the non-FH group. Examining the correlation between PS and Achilles tendon thickness yielded a correlation coefficient of 0.393 (p = 0.020), indicating a significant correlation (Figure 2A). On the other hand, no correlation was observed between IMT and Achilles tendon thickness (Figure 2B).

## Discussion

In this study, we confirmed that the FH group had significantly higher PS and IMT values than the non-FH group. Regarding the efficacy of PS and IMT in assessing carotid artery atherosclerosis and estimating cardiovascular risk, previous reports suggest that PS may be a better indicator than IMT when comparing FH and non-FH groups [4,5]. Another previous report indicated that PS is a more valuable indicator than IMT for assessing coronary artery disease risk in FH patients [9]. Although there have only been a few studies on sensitivity and specificity for predicting atherosclerosis in FH patients, one report showed that carotid plaque thickness (≥ 1.15 mm) evaluated by carotid ultrasound has high sensitivity (78.4%) and specificity (79.3%) [10]. However, there are various methods for evaluating PS and IMT, and the significance of these indicators is not always agreed upon across studies. In this study, while it is necessary to consider that the difference in IMT between the two groups was less significant than the difference in PS, both PS and IMT were suggested to be good indicators for evaluating the progression of atherosclerosis in FH.

**Figure 3:**
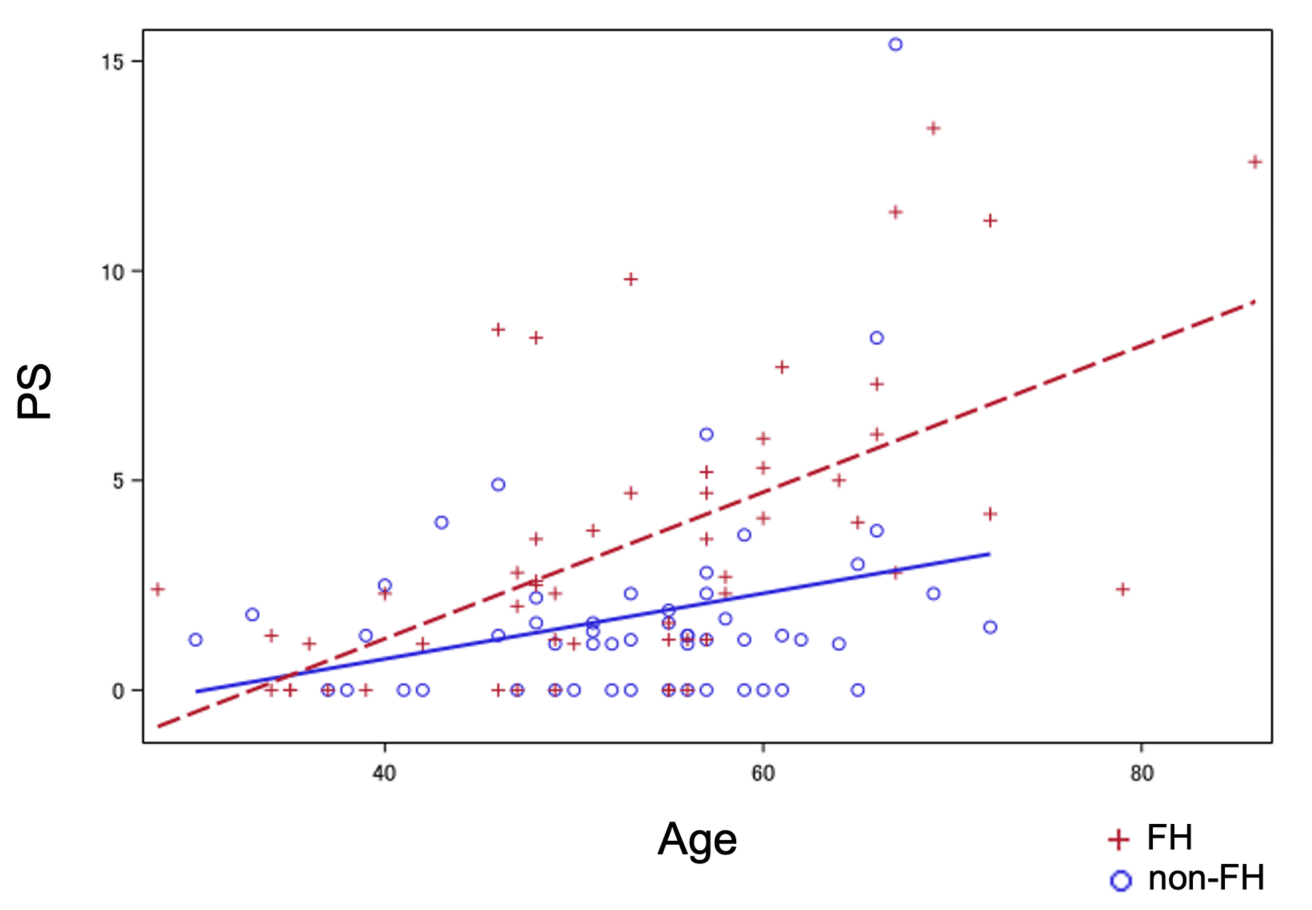
Correlation between PS and age in FH and in non-FH Scatter plot of PS vs. age in FH (n = 53) and in non-FH (n = 53). Linear regression lines for each group are also shown; the broken line represents the FH group, and the solid line represents the non-FH group. Only cases after the propensity score matching were used. PS: plaque score; FH: familial hypercholesterolemia

In age-specific subgroup analyses of PS and IMT, no significant differences were observed between the two groups in those under 50 years of age. In those aged 50 years or older, however, both PS and IMT were significantly higher in the FH group than in the non-FH group. It has been recently proposed that the progression of atherosclerotic diseases is proportional to cumulative LDL-C levels [11]. The present results suggest that, while cumulative LDL-C levels did not reach a threshold for significant differences in atherosclerotic progression between the FH and non-FH groups under 50 years of age, cumulative LDL-C levels did reach this threshold in cases where the first carotid ultrasound was performed at 50 years of age or older. Although further investigation is needed regarding the treatment and course prior to the first carotid ultrasound examination, as well as the timing of FH diagnosis, FH cases aged 50 years or older at the time of the first carotid ultrasound were likely not diagnosed with FH prior to referral to the clinic and may not have received adequate treatment beforehand. Thus, this study suggests that early diagnosis of FH is important for atherosclerosis prevention in FH. As an exploratory evaluation, scatter plots were created for age and PS, and linear regression analyses were performed; PS seemingly increases more readily with age in FH than in non-FH (Figure 3), supporting the importance of early diagnosis of FH.

Analysis of the changes and change rates in PS and IMT in cases with data from two or more carotid ultrasound examinations revealed no significant differences between the FH and non-FH groups. This suggests that, with consistent administration of lipid treatment in specialist centers, the progression of atherosclerosis in FH patients may be equivalent to that in non-FH patients. Considering that treated FH may have slower IMT progression compared to untreated FH [3], and that intensified statin therapy in FH may delay IMT progression [12], more appropriate treatment for FH is important for preventing the progression of atherosclerosis. However, age-specific subgroup analysis in this study revealed that the change in PS was significantly higher in the FH group than in the non-FH group in those under 50 years of age. Further investigation is needed to consider age-related differences in adherence, but this should be noted when treating young FH patients.

In sex-based analyses, women were found to be more strongly associated with PS values than men in the FH group. While the higher number of female cases in this study may have contributed to this result, previous reports have also indicated that factors associated with carotid atherosclerosis differ between male and female FH patients [13]. Therefore, we consider it necessary to perform regular carotid ultrasound examinations and monitoring with particular attention in female FH patients.

Interestingly, when patients were divided into groups based on LDL-C levels (≥140 mg/dL and <140 mg/dL), no significant difference in PS was observed between the FH and non-FH groups in the LDL-C ≥140 mg/dL group, whereas the PS was significantly higher in the FH group in the LDL-C <140 mg/dL group. Since the timing of the initiation of lipid-lowering treatment and its course prior to carotid artery ultrasound examination may be involved, further investigation into these aspects of each case is expected.

In FH, Achilles tendon thickness has been reported to be associated with carotid artery ultrasound findings of atherosclerosis [14,15] and LDL-C levels [16]. In the present study, a correlation was also observed between PS and Achilles tendon thickness, suggesting that more aggressive lipid management may be necessary in cases with marked Achilles tendon thickness.

In this study, the causative genes of the FH cases had largely not been investigated. FH caused by LDL receptor mutations could result in greater carotid IMT than FH caused by ApoB or PCSK9 mutations [17]. Further research is expected to shed light on the relationship between genetic mutations and carotid ultrasound findings or systemic atherosclerosis.

Limitations

Several limitations of this study should be noted. First, as this was a single-center study, biases in the characteristics of the enrolled cases or treatment strategy at the clinic may have influenced the results, so caution is required when generalizing the findings. Second, there may be uncollected confounding factors beyond those included in this study. Third, the propensity score matching reduced the number of cases included in the analyses. In particular, in the FH group, there were fewer patients with diabetes, hypertension, and a history of smoking before matching; therefore, there were fewer cases with these risk factors for atherosclerotic diseases in the matched population. Previous reports have shown that the factors predicting cardiovascular disease in FH include diabetes [18,19], hypertension, advanced age, lower HDL-C levels, male sex, and smoking [20]. Although the factors that cause atherosclerosis in FH are still a matter of debate, FH patients with different backgrounds might have different risk factors for arteriosclerosis. Further large-scale studies are needed to investigate how carotid ultrasound findings change in FH patients with these risk factors. Fourth, as we did not collect data on cumulative LDL-C levels, the relationship between cumulative LDL-C levels and changes in PS and IMT could not be examined. We also did not examine the changes in the medication, including cholesterol-lowering drugs or others such as eicosapentaenoic acid, which may indirectly influence PS and IMT. Fifth, this study focuses solely on carotid ultrasound findings between FH and non-FH, and there is still no consensus that these measurements are entirely useful as surrogate markers for predicting atherosclerosis-related mortality or assessing treatment efficacy.

## Conclusions

This retrospective observational study revealed that PS and IMT are significantly higher in the FH group than in the non-FH group. While there was no difference in PS and IMT between the FH and non-FH groups in those under 50 years of age, the PS and IMT were significantly higher in the FH group than in the non-FH group in those aged 50 years or older, suggesting the importance of early diagnosis and treatment of FH. Additionally, under appropriate lipid management, no significant differences were observed in the changes or change rates in PS and IMT between the FH and the non-FH groups, suggesting that appropriate treatment can suppress the progression of atherosclerosis in FH to the level comparable to that in non-FH.

## References

[1] Nezu T, Hosomi N, Aoki S, Matsumoto M (2016). Carotid intima-media thickness for atherosclerosis. J Atheroscler Thromb.

[2] Tada H, Nohara A, Usui S (2023). Coronary artery and carotid artery plaques in patients with heterozygous familial hypercholesterolemia. JACC Adv.

[3] van Bergen En Henegouwen K, Hutten BA, Luirink IK, Wiegman A, de Groot E, Kusters DM (2022). Intima-media thickness in treated and untreated patients with and without familial hypercholesterolemia: A systematic review and meta-analysis. J Clin Lipidol.

[4] Shibayama J, Tada H, Sakata K, Usui S, Takamura M, Kawashiri MA (2022). The assessment of carotid atherosclerotic plaque among young patients with familial hypercholesterolemia. Intern Med.

[5] Ershova AI, Balakhonova TV, Meshkov AN, Rozhkova TA, Boytsov SA (2012). Ultrasound markers that describe plaques are more sensitive than mean intima-media thickness in patients with familial hypercholesterolemia. Ultrasound Med Biol.

[6] Björkegren JL, Lusis AJ (2022). Atherosclerosis: Recent developments. Cell.

[7] Harada-Shiba M, Arai H, Oikawa S (2012). Guidelines for the management of familial hypercholesterolemia. J Atheroscler Thromb.

[8] Okamura T, Tsukamoto K, Arai H (2024). Japan Atherosclerosis Society (JAS) Guidelines for Prevention of Atherosclerotic Cardiovascular Diseases 2022. J Atheroscler Thromb.

[9] Tada H, Kawashiri MA, Okada H (2017). Assessments of carotid artery plaque burden in patients with familial hypercholesterolemia. Am J Cardiol.

[10] Deniz MF, Guven B, Ebeoglu AO (2025). Screening for subclinical atherosclerosis in patients with familial hypercholesterolemia: Insights and implications. J Clin Med.

[11] Ference BA, Braunwald E, Catapano AL (2024). The LDL cumulative exposure hypothesis: Evidence and practical applications. Nat Rev Cardiol.

[12] Vergeer M, Zhou R, Bots ML (2010). Carotid atherosclerosis progression in familial hypercholesterolemia patients: A pooled analysis of the ASAP, ENHANCE, RADIANCE 1, and CAPTIVATE studies. Circ Cardiovasc Imaging.

[13] Waluś-Miarka M, Czarnecka D, Kloch-Badełek M, Wojciechowska W, Kapusta M, Malecki MT (2017). Carotid artery plaques - Are risk factors the same in men and women with familial hypercholesterolemia?. Int J Cardiol.

[14] Kiortsis DN, Argyropoulou MI, Xydis V, Tsouli SG, Elisaf MS (2006). Correlation of Achilles tendon thickness evaluated by ultrasonography with carotid intima-media thickness in patients with familial hypercholesterolemia. Atherosclerosis.

[15] Jarauta E, Junyent M, Gilabert R (2009). Sonographic evaluation of Achilles tendons and carotid atherosclerosis in familial hypercholesterolemia. Atherosclerosis.

[16] Ogura M, Harada-Shiba M, Masuda D (2022). Factors associated with carotid atherosclerosis and achilles tendon thickness in Japanese patients with familial hypercholesterolemia: A subanalysis of the familial hypercholesterolemia expert forum (FAME) study. J Atheroscler Thromb.

[17] Bekbossynova M, Ivanova-Razumova T, Azatov Y, Sailybayeva A, Khamitov S, Daniyarova G, Akzholova K (2025). Genetic variants and carotid atherosclerosis progression in familial hypercholesterolemia: A comprehensive review. Front Cardiovasc Med.

[18] Boutari C, Rizos CV, Doumas M (2023). Prevalence of diabetes and its association with atherosclerotic cardiovascular disease risk in patients with familial hypercholesterolemia: An analysis from the Hellenic familial hypercholesterolemia registry (HELLAS-FH). Pharmaceuticals (Basel).

[19] Paquette M, Cariou B, Bernard S (2024). Increased FH-risk-score and diabetes are cardiovascular risk equivalents in heterozygous familial hypercholesterolemia. Arterioscler Thromb Vasc Biol.

[20] Paquette M, Dufour R, Baass A (2017). The Montreal-FH-SCORE: A new score to predict cardiovascular events in familial hypercholesterolemia. J Clin Lipidol.

